# Impact of a New Nasal Pillows Mask on Patients' Acceptance, Compliance, and Willingness to Remain on CPAP Therapy

**DOI:** 10.1155/2016/6713236

**Published:** 2016-08-25

**Authors:** Alison Wimms, Sahisha Ketheeswaran, Claus Ziegenbein, Laura Jennings, Holger Woehrle

**Affiliations:** ^1^ResMed Science Centre, Fraunhoferstraße 16, 82152 Martinsried, Germany; ^2^Sleep and Ventilation Center Blaubeuren, Lung Center Ulm, Ulm, Germany

## Abstract

*Aim*. Continuous positive airway pressure (CPAP) masks are a key factor in patient compliance. This program assessed the performance of a new nasal pillows mask (NPM) on a variety of new and established obstructive sleep apnea (OSA) patients using CPAP therapy.* Methods*. Five programs were developed to assess the new NPM [AirFit P10, ResMed] on naïve patients; patients established on another NPM; patients using a nasal mask; patients with low CPAP compliance; and patients who wished to stop using CPAP therapy.* Results*. A total of 212 patients were included. In naïve patients, CPAP usage after 3 months was 5.9 ± 1.7 hours/night, compared with the control group at 4.6 ± 2.4 hours/night (*p* < 0.05). In patients established on another NPM, usage improved to 7.4 ± 1.1 hours/night versus 6.7 ± 1.4 (*p* = 0.001). 78% of nasal mask users wished to continue using the new NPM. Low compliance patients improved with an average of 0.87 hours/night (*p* = 0.001) when using the new NPM. In patients at the point of quitting CPAP, 60% continued with therapy using the new NPM*. Conclusion*. The new NPM mask performed well in a variety of clinical groups of OSA patients receiving CPAP therapy and shows that technical advances in CPAP masks can improve patient compliance.

## 1. Introduction

Obstructive sleep apnea (OSA) is a major health problem. It is known to cause excessive daytime sleepiness, motor vehicle accidents, impaired cognitive function, and reduced quality of life [1]. It has also been associated with serious health consequences such as hypertension, cardiovascular morbidity and mortality, stroke, and diabetes [1]. Effective treatment with continuous positive airway pressure (CPAP) has been shown to improve symptoms and reduce health risks in these patients [2].

Despite the effectiveness and known benefits of CPAP, compliance with therapy (generally defined as >4 hours' usage on 70% of nights) [3] remains challenging, with adherence rates ranging from 30 to 74% [4, 5]. Although low compliance is an ongoing challenge for CPAP users and respiratory services, of even more concern is that >30% of patients will withdraw from positive airway pressure (PAP) therapy entirely [2, 6].

Adherence to PAP therapy is key to ensuring its success, with research showing that >4 hours' usage may be required to improve blood pressure [7–9], usage for >6 hours/night may be needed to effect improvements in cognitive function [10, 11], and, in general, greater use is associated with greater benefits, particularly regarding daytime sleepiness [12, 13].

The ability of CPAP users to find an interface that suits them is one of the main determinants of acceptance and adherence to CPAP and healthcare utilization [14, 15]. The three main mask types available are full face masks (also known as oronasal masks), which cover the nose and mouth; nasal masks, which cover the nose only; and nasal pillows masks (NPMs), which apply CPAP air directly to the nares via cushions applied at the nostrils. Although the technology and design of interfaces have evolved significantly over time, finding a compatible interface remains an important, but challenging, task for CPAP users. Common interface-related side effects include discomfort, pressure sores, unintentional mask leak, skin reactions, and claustrophobia [3, 16]. The development of NPMs provides an option that decreases these side effects through a smaller contact surface area with the face, less pressure applied on the nose while also avoiding any pressure on the nasal bridge, and reduced obtrusiveness [17].

Very few studies have investigated the impact of different mask types on patient outcomes. Small studies have reported that NPMs are more comfortable, have fewer side effects, and improve overall satisfaction with CPAP [18, 19]. NPMs have also been associated with significantly better compliance (expressed as the percentage of days of CPAP use) [18]. Furthermore, it has been noted that the design of NPMs may mean that users report less air leak into their eyes [20]. A Cochrane review of available data suggested that NPMs may be a useful alternative for patients unable to tolerate conventional nasal masks but recommended that further trials be conducted [17]. A recently published study identified differences in compliance with CPAP when nasal masks from different manufacturers were used, with initial mask choice influencing adherence and healthcare utilization over a 3-month period [15].

This report details the results of a program that investigated the use of a new NPM. The mask [AirFit P10; ResMed] was designed to be small and unobtrusive, and advances in technology mean it is approximately 50% lighter than its predecessor [Swift Fx, ResMed]. The mask also contains a mesh woven vent which aims to significantly reduce noise and venting disturbance. The impact of the new NPM on compliance and acceptance was investigated in a variety of new and established CPAP users.

## 2. Materials and Methods

Use of a new NPM [AirFit P10; ResMed] was assessed in several different groups of CPAP users across Australia, Germany, and the United Kingdom: new CPAP users (Group 1); CPAP users established on a different model of NPM (Group 2); CPAP users established on a nasal mask (Group 3); patients with low compliance to CPAP therapy (Group 4); and patients wishing to stop CPAP (Group 5). Where the treatment strategy differed from routine clinical practice, studies were approved by the local ethics committees. All patients provided informed consent before inclusion in the study, and all experiments were conducted in accordance with Good Clinical Practice and the principles of the Declaration of Helsinki.

### 2.1. New CPAP Users (Group 1)

CPAP-naïve patients with OSA were set up with the new NPM at a Sleep Lab in Ulm, Germany, between April 2014 and December 2014. For the first three months of treatment patients were monitored for adherence, mask changes, and therapy withdrawal. In addition, clinicians involved in the trial were asked how easy it was to set up the new NPM mask compared with other NPMs and nasal masks. Participants who changed masks during the first three months were assessed (excluding those who changed to a full face mask due to persistent mouth breathing). Results were compared with a group of patients who started therapy with a nasal mask over the same period (nasal masks were from a variety of manufacturers). Average daily usage difference between the two groups was compared using the paired* t*-test. The aim of this comparison was to assess whether the new NPM could be used successfully in patients new to CPAP treatment.

### 2.2. Established NPM Users (Group 2)

OSA patients being managed at the ResSleep Centre, Sydney, Australia, and using an older style of NPM [Swift Fx, ResMed] were asked to take home the new NPM for a one-week trial to assess performance compared with their current NPM. During the initial consultation, objective baseline CPAP usage data (usage hours, apnea-hypopnea index [AHI], pressure and leak) from seven consecutive nights preceding new NPM trial were downloaded from the participants' CPAP devices. Participants then used the new NPM for seven consecutive nights. At the end of the seven-night study period, participants completed questionnaires to provide feedback on the performance of the new NPM compared with their usual NPM, as well as overall mask preference. For each category participants rated the mask using an 11-point Likert Scale where 0 means very poor performance and 10 means excellent performance. Scores were compared between the new NPM and the participants' usual mask using the Wilcoxon signed ranks test. CPAP usage data were again downloaded from the CPAP device after the seven-night mask trial and compared to the participants' usual mask CPAP data using the paired* t*-test.

### 2.3. Established Nasal Mask Users (Group 3)

CPAP users already established on a nasal mask at a range of hospitals in the UK, who contacted their local care team for a routine query or part replacement, were asked if they would like to trial a NPM in place of their regular nasal mask. Participants who agreed were provided with the new NPM to use for their routine CPAP. After two months' use of the new NPM, participants were contacted to complete a 7-point Likert questionnaire where 1 means poor performance and 7 means excellent performance. Data is displayed in terms of median scores for each mask (Figure 2). The goal was to assess how the new NPM rated compared with the existing nasal mask and to see if patients established on a nasal mask would be willing to switch to a NPM.

### 2.4. CPAP Users with Low Compliance (Group 4)

Patients using any brand of nasal mask or NPM with low usage (<5 hours/night) who were monitored by ResMed Germany Healthcare, a German homecare provider, were identified via wireless monitoring (AirView, ResMed). Patients were contacted by healthcare staff and asked if they would be willing to trial a different mask. Participants who agreed were posted the new NPM. Compliance with CPAP therapy after patients changed to the new NPM was monitored on an ongoing basis using wireless monitoring to determine the impact of the new NPM on compliance in patients with low usage. Data before and after the mask change was analysed using the McNemar Test.

### 2.5. Patients Wanting to Quit CPAP Therapy (Group 5)

ResMed Germany Healthcare manages a large cohort of patients being treated with PAP therapy. As with all homecare companies, a proportion of patients phone the provider every month wanting to quit PAP therapy. Approximately 15% of these patients cite mask issues as a reason for wanting to stop using CPAP. For this study, patients who phoned the homecare provider wishing to quit therapy who mentioned mask-related issues were informed about the new NPM; they were told that a new mask was available that might potentially help improve their experiences with CPAP. Subjects were offered a two-week trial of the new NPM before they made a final decision to quit CPAP therapy. Those who agreed to this were posted the NPM and followed up after two weeks and eight months to determine CPAP usage. Data is displayed via a consort diagram (Figure 3).

## 3. Results 

A total of 212 patients were included, 46, 21, 27, 64, and 54 in Groups 1, 2, 3, 4, and 5, respectively.

### 3.1. New CPAP Users

Forty-six CPAP-naïve patients were set up using the new NPM (age 53 ± 10 years, 74% male, body mass index [BMI] 32 ± 8 kg/m^2^, Epworth Sleepiness Scale [ESS] score 10 ± 5, and apnea-hypopnea index [AHI] 43 ± 18). Two patients rejected CPAP therapy at the time of equipment set-up and did not proceed. After successful set-up in 44 patients, 2 stopped therapy, and 5 switched to another mask during the 3-month follow-up period. After 3 months, average daily CPAP usage in the new NPM group was 5.9 ± 1.7 hours/night; corresponding usage in the control group who used a nasal mask as the first CPAP interface was 4.6 ± 2.4 hours/night (*p* < 0.05). The proportion of patients who stopped CPAP was 4.5% in the new NPM group and 3% in the control group. In the new NPM group, 2% of participants changed to a nasal mask compared with 13% in the control group. All of the of clinicians interviewed felt they spent less time with the patient at the first visit showing them how to use the new NPM, and all felt that the new NPM leaked less than other NPMs.

### 3.2. Established NPM Users

**Figure 1:**
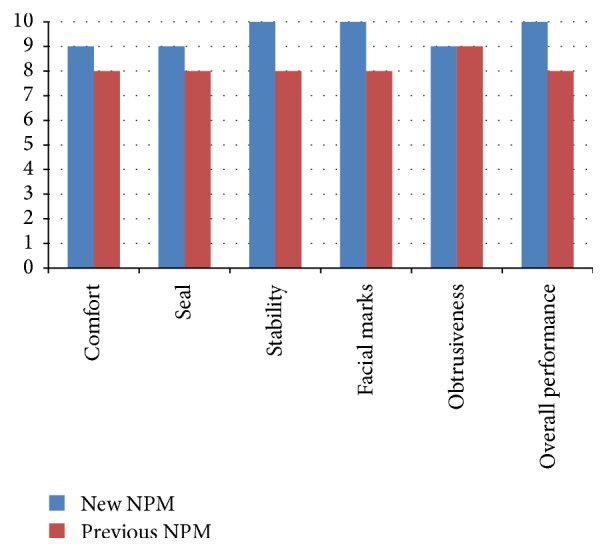
Average scores of participants asked to rate the new NPM compared with their current NPM (Group 2). Participants were asked to rate each aspect of the mask using an 11-point Likert Scale where 0 means poor performance and 10 means excellent performance.

The 21 patients already established on NPMs had an average age of 61 ± 11 years and 81% were male. After trialing the new NPM for 7 consecutive nights, patients rated it as superior to their previous NPM in overall seal, stability, overall performance, vent flow and noise for the patient, vent flow and noise for the bed partner, and overall performance (*p* < 0.05) (Figure 1). CPAP device data showed that there was a significant difference in average daily usage hours between the new and previous NPM: 7.4 ± 1.1 for the new NPM versus 6.7 ± 1.4 for the previous NPM (*p* = 0.001). There were no significant differences between the two NPMs for all other downloaded data (mask leak, pressure, and AHI).

### 3.3. Established Nasal Mask Users

**Figure 2:**
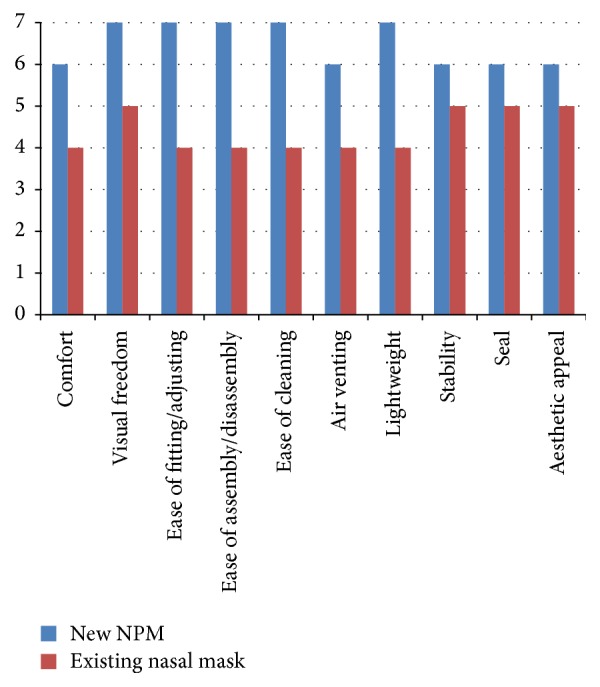
Median scores of participants asked to rate the new NPM compared with their current nasal mask (Group 3). Participants were asked to rate each aspect of the mask using a 7-point Likert Scale where 1 means poor performance and 7 means excellent performance.

The new NPM scored higher than the patient's previous nasal mask on each category assessed (Figure 2). The overall rating for the new NPM was 6.1/7 compared with 4/7 for the previous nasal mask. In total, 67% of patients felt that the new NPM made using CPAP therapy easier. Overall, 78% of those who trialed the new NPM wanted to continue using it rather than returning to their previous nasal mask.

### 3.4. Low Compliance Users

All 64 patients identified as having low CPAP usage agreed to trial the new NPM. Average daily usage significantly improved from 2.25 hours on the previous mask to 3.12 hours on the new NPM (*p* = 0.001). AHI and leak also improved after switching to the new NPM, but changes compared with the previous interface did not reach statistical significance. Of patients previously considered noncompliant with CPAP, 14% achieved the threshold to be defined as compliant (average usage ≥4 hours/night) when using the new NPM.

### 3.5. Patients Wanting to Quit CPAP

**Figure 3:**
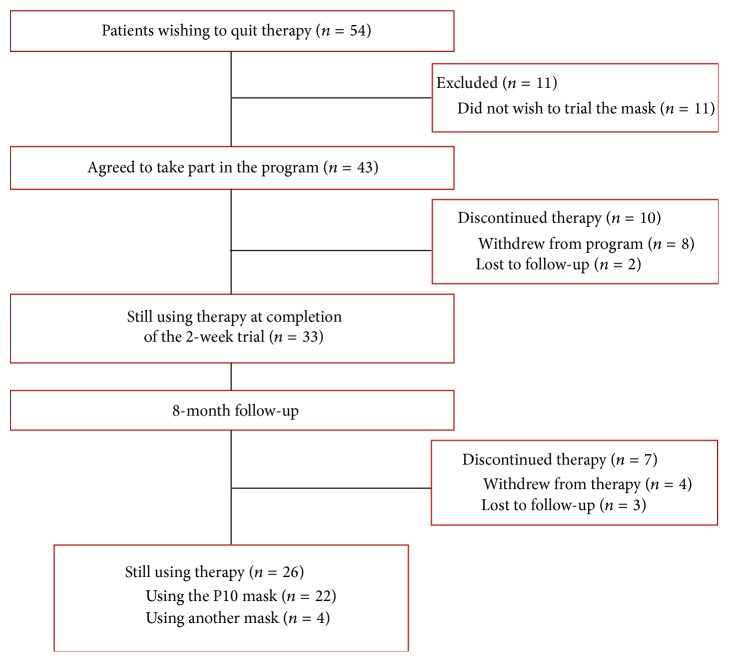
Results of program where patients wishing to quit therapy were offered a two-week trial of the new NPM (Group 5).

Of the 54 patients who wanted to quit therapy (age 59.8 ± 11.5 years, 69% male), 43 (80%) were willing to trial the new NPM for two weeks. When contacted by phone after 2 weeks, 33/44 patients (77%) were using the new NPM nightly and wanted to continue CPAP with the new NPM. At the 8-month follow-up, 26/43 patients (60%) who agreed to trial the NPM were still using CPAP therapy (Figure 3).

## 4. Discussion

Initial mask selection is a key factor for influencing CPAP adherence and utilization of healthcare services. Typically, CPAP-naïve patients are provided with nasal masks at treatment initiation. During this program the new NPM was suitable as a first interface for new CPAP users and resulted in fewer mask changes and better compliance during the first three months of therapy compared with a matched group who started therapy with a nasal mask. NPMs are not usually regarded as first-line CPAP interfaces and may be reserved for patients who experience claustrophobia or who are unable to tolerate nasal masks. This might be due to concerns regarding the performance of nasal pillows at high CPAP pressures. It is commonly thought that NPMs may have inadequate fit, seal, and effectiveness and may increase the occurrence of side effects. However, studies have shown that there is no increase in side effects for new or experienced patients using NPM, even at high CPAP pressures (≥12 cm H_2_O) [19, 20], and actually NPMs may have advantages, such as reduced pressure on the face, less obtrusiveness, and less claustrophobia, which may be preferable for new users [19, 20]. This program shows that NPMs may be a better choice for new patients, resulting in better compliance and fewer mask changes.

We also found that a trial of the new NPM in patients established on older NPMs or nasal masks was associated with higher subjective satisfaction and increased compliance with CPAP therapy. Each iteration of a new mask aims to overcome limitations of previous designs and provide a more comfortable, easier to use, less obtrusive interface for users. Previous studies have found that advances in technology can improve compliance [21]; however none have shown this improvement with a change of mask. Considering that longer hours of CPAP use are associated with increased benefits [12, 13], an increase in compliance at any stage should be considered beneficial. Our findings support the hypothesis that NPMs may have benefits over nasal masks due to their smaller size and less obtrusive nature. Despite these potential benefits of NPMs, other factors such as system noise and bed partner disturbance from air venting may still persist as issues, although these were not specifically raised as concerns during our programs [22, 23].

Finally, in the most challenging patients (those with low compliance or who want to stop CPAP therapy), we found that use of the new NPM as the CPAP interface was associated with an increase in compliance and a willingness to continue with therapy. This suggests that an intervention as simple as a mask change alone can improve compliance with PAP therapy and that most patients who express a desire to quit PAP therapy are actually looking for solutions to existing problems rather than reasons to quit, because most were willing to trial another interface. Furthermore, most of those who trialed the new NPM were able to successfully continue with therapy. This shows that a well-fitting, comfortable mask can keep patients who were at the point of withdrawing from therapy adherent to PAP treatment.

In addition to the individual benefits seen with continuing use of CPAP, health economic benefits must also be considered. It has been estimated that untreated OSA patients in Europe have a 2-fold increase in medical expenses [24]. When considering the effects of the disease on fatigue, cognitive functioning, workplace performance, occupational injury, and vehicle accidents, the socioeconomic costs are estimated to be immense, running into the billions [25]. Treating OSA with PAP has shown to decrease these excessive medical costs and is a cost-effective way to manage the disease [24–26]. When a patient who has been initiated on CPAP then withdraws from therapy, the investment taken to test and treat the patient, including physician visits and CPAP equipment, is lost. Therefore, low cost programs such as this aimed at keeping participants on therapy should be utilized as much as possible. This study had several limitations. The use of different patient groups across different clinics is a potential source of bias because of different clinical practices at each center. In addition, there were no prospective control groups, and the matched control groups included were being treated with a variety of masks from different manufacturers, which may have influenced results. Finally, our assessments were designed in a pragmatic manner to fit in with clinical practice and therefore should be investigated further in studies with a more rigorous design.

## 5. Conclusion

This series of investigations found that a new NPM mask performed well in a variety of clinical groups of OSA patients receiving CPAP therapy. The use of a new NPM facilitates good acceptance of CPAP therapy in new users, improved compliance in existing users and those struggling with therapy, and facilitated continuation with CPAP in patients who were at the point of quitting therapy. Effective use of CPAP is important to reduce the burden of OSA and associated comorbidities, at both the healthcare system and individual levels. Therefore, there is a need for CPAP masks being able to optimize users' acceptance of therapy. Technological advancements have, and will continue to play, an integral role in the design of better and more user-friendly interfaces, which can improve patient acceptance of, and compliance with, CPAP therapy.
